# Lysolecithins improved growth performance, nutrient digestibility, immunity, and antioxidant ability in broiler chickens

**DOI:** 10.5713/ab.23.0442

**Published:** 2024-02-23

**Authors:** Yuanli Cai, Lu Gao, Bochen Song, Zhigang Song

**Affiliations:** 1 College of Life Science, Qilu Normal University, Jinan, Shandong 250200, China; 2 Key Laboratory of Efficient Utilization of Non-grain Feed Resources, College of Animal Science and Technology, Shandong Agricultural University, Taian, Shandong 271018, China

**Keywords:** Broiler Chicken, Immunity, Liver Health, Lysolecithins

## Abstract

**Objective:**

This study investigated the effects of dietary supplementation with lysolecithins (LPC) on growth performance, nutrient digestibility, blood profiles, immunity, and liver health in broiler chickens.

**Methods:**

A cohort of 240 one-day-old male Arbor Acres broilers of comparable weight was divided into four treatment groups, each comprising six replicates of 10 birds. The groups were defined as follows: positive control with recommended metabolizable energy (PC+ME), negative control with 90 kcal/kg reduced ME (NC+ME), PC supplemented with 300 mg/kg LPC (PC+LPC), and NC supplemented with 300 mg/kg LPC (NC+LPC).

**Results:**

LPC supplementation led to a statistically significant reduction in the feed conversion ratio (p = 0.05) and a decrease in the proportion of abdominal fat and the liver (p<0.05). Digestibility of dry matter was also enhanced (p<0.05). Malondialdehyde concentrations in the liver were significantly reduced by LPC (p<0.01), with a noteworthy interaction between energy levels and LPC affecting this reduction (p<0.05). Serum levels of interleukin-6 were reduced on day 21, and both endotoxin and interleukin-6 levels were lower on day 42. Notably, a significant interaction was observed between the energy levels and LPC on relative liver weight and endotoxin concentrations in the serum (p<0.05).

**Conclusion:**

The study concluded that LPC positively affects growth performance, nutrient digestibility, immune response, and antioxidative capacity in broiler chickens, affirming its value as a beneficial feed additive in poultry nutrition.

## INTRODUCTION

In contemporary poultry production, augmenting fat content is a prevailing strategy to meet the heightened energy requirements of broilers, as fat has the highest caloric value among all essential nutrients. However, the incorporation of fat results in increased feed expenses. Furthermore, challenges arise in the digestion and utilization of lipids in broilers. In juvenile broilers, lipid absorption and digestion are insufficient because of incomplete development of the digestive tract, which fails to generate and secrete bile salts and lipase in adequate quantities [1]. To overcome the physiological constraints on lipid utilization in broiler chickens, the use of exogenous emulsifiers, such as bile salts and lysolecithin (LPC), has emerged as a viable solution. LPC, also known as lysophosphatidylcholine, originates from the hydrolysis of phospholipids and functions as an efficient emulsifying agent. The inclusion of dietary LPC effectively reduces the size of fat globules and augments the active surface area for lipase digestion [2].

Dietary supplementation with LPC has been shown to enhance nutrient and energy utilization in young broilers [3]. Jansen et al [4] similarly observed that the incorporation of LPC into feed formulations can elevate digestibility and energy values, particularly when incorporating saturated fat sources. LPC supplementation has been associated with increased body weight gain (BWG) in broiler chickens during the starter period, potentially because of the enhanced coefficient of total tract apparent digestibility of fatty acids due to LPC activity [5]. However, in contrast to the aforementioned research, some studies have reported that digestibility of dry matter (DM), crude protein (CP) and fat were not effected by LPC addition to a low energy diet [6]. The addition of emulsifiers to diets with low energy density did not substantially influence broiler growth performance [7,8].

Assessments of the effects of distinct emulsifiers on broiler chickens are limited and inconsistent. Furthermore, there is a dearth of research exploring the effects of supplemental LPC on the immune response of broiler chickens. Consequently, the objective of the present study was to scrutinize the effects of LPC supplementation in low-energy diets on the growth performance, nutrient digestibility, blood profiles, immunity, and liver health of broiler chickens.

## MATERIALS AND METHODS

### Experimental design and management

The protocol for the current experiment was received and approved by the Institutional Animal Care and Use Committee at Shandong Agriculture University (IACUC:0026-1896).

A total of 240 male, one-day-old Arbor Acres broilers (purchased from Dabao Breeding Company, Shandong, China) with similar initial weights (42±0.5 g) were divided into four groups and subjected to various dietary regimens. Each group consisted of six replicates, each containing 10 chickens. The four dietary treatments were as follows: positive control with recommended metabolizable energy (PC+ ME), negative control with 90 kcal/kg reduced ME (NC+ME), PC supplemented with 300 mg/kg LPC (PC+LPC), and NC supplemented with 300 mg/kg LPC (NC+LPC). Experimental LPC was sourced from Guangdong Hinabiotech Co., Ltd. (Guangdong, China). The formulation of the experimental diet, detailed in Table 1, adhered to the National Standard of the People’s Republic of China titled “Compound Feed for Laying Chickens and Broilers’ (GB/T 5916-2020). The feeding regimen comprised two distinct phases: the starter phase (days 1 to 21) and finisher phase (days 22 to 42). The broilers were raised in an environmentally controlled room with the cages of 0.5 square meters. The brooding temperature was maintained at 35°C for the first 2 d, and then reduced gradually to 21°C until 28 days of age, where it was maintained until the end of the experiment. The light regime was 23 hours of light (from 01:00 to 24:00 h) and 1 hour of darkness (from 24:00 to 01:00 h). The ambient relative humidity was 40% to 50%. Both feed and water were provided *ad libitum*.

### Performance measurements

Body weight (BW) and feed intake (FI) of broiler chickens were assessed on day 1, 21, and 42 of the experiment. These data were employed to compute the following parameters for each time interval: average daily gain (ADG), average daily FI, and feed conversion ratio (FCR).

### Nutrient digestibility

Nutrient digestibility of the ileum was assessed using the acid-insoluble ash (AIA) marker method, following the methodology outlined by McCarthy et al [9]. In this approach, diets were supplemented with 0.5% AIA as an indigestible marker, and broilers were provided with these modified diets during the final four days of the trial. On day 42, two chickens were randomly selected from each enclosure and euthanized via cervical dislocation. Subsequently, the ileal contents, specifically the portion extending from 5 cm behind the Meckel’s diverticulum to 4 cm before the ileocecal junction, were collected directly into containers. The digesta samples were promptly frozen at −20°C for subsequent analysis.

The freeze-dried digesta and feed samples were ground and analyzed for AIA, CP, ether extract (EE), acid detergent fiber (ADF), and neutral detergent fiber (NDF), in accordance with the protocols outlined by the Association of Official Analytical Chemists (2002). Nutrient digestibility was calculated based on the ratio of AIA to sample content.

### Carcass and part yields

On day 42, two broiler chickens were randomly chosen from each enclosure, individually weighed with ten hours feed restriction, and euthanized via cervical dislocation. After the slaughter and bleeding procedures, the carcasses were thoroughly defeathered and eviscerated. The weight of each carcass was then recorded. Additionally, abdominal fat and total liver were collected, weighed, and expressed as a percentage relative to the carcass weight. Carcass yield was calculated as the percentage of carcass weight relative to the BW.

### Sample collection

On days 21 and 42, one healthy broiler chicken with a comparable BW was chosen from each replicate. Blood was collected through the sinus of the sub-wing vein, and the serum was carefully separated and preserved at −20°C for subsequent analysis. Tissue samples were procured from the liver, washed with ice-cold sterilized saline, rapidly cooled in liquid nitrogen, and stored at −80°C until further analysis.

### Serum and liver analysis

The concentrations of glucose (GLU), triglyceride (TG), and total cholesterol (TC) were determined using a fully automated biochemical analyzer (7020; Hitachi, Co., Ltd., Tokyo, Japan). Non-esterified fatty acids (NEFA; No. A042) were analyzed using spectrophotometric techniques employing colorimetric enzymatic methods, utilizing commercial diagnostic kits from Jiancheng Bioengineering Institute (Nanjing, China). The immune response status in the serum and liver was evaluated by measuring the levels of interleukin 1β (IL-1β; No. ml059835), and interleukin 6 (IL-6; No. ml059839), endotoxins (ET; no. ml059937), immunoglobulin A (IgA; no. ml002792), and immunoglobulin G (IgG; no. ml042771) using enzyme-linked immunosorbent assay kits from MLBIO Co., Ltd. (Shanghai, China).

The aspartate aminotransferase (AST, No. ml060767), and alanine aminotransferase (ALT, No. ml060766) levels were determined using the enzyme-linked immunosorbent assay kits provided by MLBIO Co., Ltd. (Shanghai, China). Serum catalase (CAT; No. A007-1-1), superoxide dismutase (SOD; no. A001-1-1), glutathione peroxidase (GSH-Px; No. A005-1-2), and malondialdehyde (MDA; no. A003-1-2) kits from Nanjing Jiancheng Bioengineering Institute, China, were used. All the procedures were performed according to the instructions provided in the respective operating manuals.

### Statistical analysis

The data were analyzed using two-way analysis of variance (ANOVA) (least significant difference) conducted with SPSS software (version 26.0). Subsequently, ANOVA was performed using Duncan’s multiple comparison test. The results are presented as treatment means accompanied by standard errors. A significance level of p<0.05 was regarded as statistically significant, while values falling within the range of 0.05≤p<0.10 were categorized as indicative of a trend.

## RESULTS

### Growth performance

The performance of the broiler chickens fed the experimental diets is summarized in Table 2. Notably, the BW at day 21 and the average ADG from days 1 to 21 exhibited a tendency towards higher values in broilers fed the normal diet than in those fed the reduced energy diet (p = 0.07, p = 0.08). Over the period from days 1 to 21, broilers receiving the basal diets demonstrated a significantly lower FCR than those on the reduced energy diets (p<0.05). Furthermore, broilers supplemented with LPC exhibited a lower FCR from days 1 to 42 than those without LPC supplementation (p = 0.05). In contrast, BW on day 42, ADG from days 22 to 42, and ADG from d1 to d 42 remained unaffected by variations in energy content or the presence of LPC in the diets (p>0.05). No significant interaction was observed between energy content and LPC with respect to BW, ADG, or FCR at any of the experimental time intervals (p>0.05).

### Nutrient digestibility

Nutrient digestibility data for DM, CP, EE, ADF, and NDF on day 42 are presented in Table 3. Notably, dietary treatments did not exert significant effects on the digestibility of EE, ADF, or NDF (p>0.05). However, the inclusion of LPC had a positive effect on DM digestibility (p<0.05) and demonstrated a notable trend toward improved CP digestibility (p = 0.065). Importantly, no interaction was observed between variations in energy content and the presence of LPC concerning nutrient digestibility throughout the course of this experiment.

### Relative organ weight and carcass weight

Table 4 presents the effects of LPC supplementation to the reduced ME diet on the relative organ and carcass weights of broiler chickens on the 42nd day. A discernible trend was observed, indicating a reduction in the proportion of abdominal fat and liver in birds receiving diets with lower ME contents (p = 0.083, p = 0.065). Interestingly, the incorporation of LPC exhibited a notable tendency towards increasing carcass weight (p = 0.083), and its addition resulted in a significant decrease in the proportion of abdominal adipose tissue and liver (p<0.05). Furthermore, a noteworthy interaction between variations in energy content and the presence of LPC was evident with respect to the relative liver weight (p<0.05).

### Blood biochemical attribute

Table 5 shows that the serum concentrations of GLU, TG, TC, and AST on day 21 remained unaffected by variations in energy content or the presence of LPC in the diets (p> 0.05). However, the concentrations of NEFA and ALT were notably reduced by the inclusion of LPC (p<0.05). Notably, a significant interaction between energy content and LPC was observed with respect to ALT concentration (p<0.01).

The effects of LPC supplementation to the reduced ME diet on serum parameters in broiler chickens at d 42 are summarized in Table 6. Specifically, the concentrations of GLU, TC, and ALT were not influenced by dietary energy levels or by the presence of LPC (p>0.05). However, there was a decrease in the concentrations of TG and AST in broiler chickens that were fed diets with reduced energy (p = 0.055, p = 0.040). Furthermore, the dietary inclusion of LPC led to a decrease in TG and NEFA concentrations (p<0.05). Importantly, no interaction between the variations in energy content and LPC was detected in the serum profiles on day 42.

### Hepatic lipid metabolism and antioxidation indicators

Table 7 presents data indicating that on day 42, the liver concentrations of TC, SOD, GSH-Px, and CAT were not influenced by variations in energy content or the presence of LPC in the diets (p>0.05). However, the inclusion of LPC resulted in a significant reduction in MDA concentration in the liver (p< 0.01). Moreover, a noteworthy interaction between energy content and LPC was evident regarding MDA concentration in the liver (p<0.05), signifying that the effect of LPC on MDA levels was more pronounced in broilers fed low-ME diets.

### Immune function

Table 8 presents the serum immune indicators in 21-day-old broiler chickens. Notably, immune indicators, such as ET, IgA, IgG, IL-1β, and IL-6, remained unaffected by variations in energy content or the presence of LPC in the diets (p>0.05). However, an interaction was observed between energy content and LPC concerning ET, IgA, and IL-6 levels in the liver (p = 0.090, p = 0.077, p = 0.047), indicating that the effect of LPC on these immune responses was more prominent in broilers fed low-energy diets.

Table 9 provides insights into the serum immune indicators in 42-day-old broiler chickens. A noteworthy reduction in ET and increase in IgA were observed in broilers that received diets featuring lower energy levels (p<0.05). Furthermore, LPC supplementation led to a significant decrease in the serum concentrations of ET and IL-6 (p<0.05). Importantly, there was a significant interaction between energy content and LPC with respect to ET concentration in the serum (p<0.05), suggesting that the influence of LPC on ET levels was more pronounced in broilers fed normal-ME diets.

## DISCUSSION

### Growth performance

Energy plays a pivotal role in the health and production of poultry. Previous studies have underscored the positive effects of high-energy diets on broilers. For instance, Niu et al [10] reported increased BWG and FI from days 1 to 21 in broilers fed high-energy diets, accompanied by a reduction in FCR. Ge et al [11] also observed that broilers fed low-energy diets gained less weight than those fed high-energy diets. This aligns with our findings, where reduced energy diet treatments led to lower BW on day 21 and lower ADG during the 1 to 21 d period, alongside higher FCR compared to basal diet treatments.

LPC, a natural surfactant derived from hydrolyzed soy lecithin, possesses a single mole of fatty acids per molecule, which enhances its hydrophilic properties. Consequently, LPC is a superior biosurfactant, particularly in terms of emulsifying properties within the aqueous environment of the gastrointestinal tract [12]. The inclusion of LPC has potential benefits for commercial broiler chickens. Several studies have explored the use of emulsifiers in low-energy diets as a nutritional strategy to reduce feed costs, without compromising growth performance. In our study, LPC supplementation improved the FCR from days 1 to 42. The reduction of FCR was related to the improvement in nutrient digestiblity [4]. However, the BW and ADG at various experimental periods were not significantly affected by the addition of LPC.

These findings align with those of Khonyoung et al [13] who reported no significant difference in bird BWG between the emulsifier and control treatments but observed an improvement in FCR in the emulsifier group. In contrast, Zhang et al [5] found that the supplementation of LPC at 0.5 g/kg improved weight gain and feed efficiency in young broilers from 1 to 21 d of age. Additionally, Boontiam et al [14] demonstrated that the addition of LPC to low-energy diets at inclusion levels of 0.10% and 0.15% leads to increased BW and BW gain. Variability in growth performance may be attributed to differences in the levels and sources of dietary fat. Furthermore, Øverland et al [15] suggested that incorporating animal fats may enhance the positive effects of emulsifiers on growth performance. In the present study, soybean oil was included in the diets, which may explain why LPC supplementation did not significantly affect BW or ADG.

### Nutrient digestibility

In the current study, the inclusion of LPC resulted in a notable improvement in the apparent digestibility of DM, and there was an observable increasing trend in the apparent digestibility of CP with LPC supplementation. These findings are consistent with those of the previous studies. Jansen et al [4] noted that the addition of LPC enhanced the digestibility of DM and improved nitrogen retention. Similarly, Zhao et al [16] observed an increase in the apparent total tract digestibility of DM and nitrogen in broilers on day 14 owing to lysophospholipid supplementation. LPC is known to enhance emulsification and digestion of fats and oils. Furthermore, the lipids present in chyme can influence the absorption of other nutrients, potentially hindering their absorption. Therefore, improved emulsification of fats by LPC can facilitate the digestion of other nutrients [17]. This is likely why LPC improved the DM digestibility.

However, despite being an effective emulsifier, LPC supplementation did not significantly affect the apparent digestibility of EE in the present study. These results align with those of previous reports, which indicated that while there were no notable differences in crude fat digestibility, the addition of LPC to pig lard feed led to a significant increase in DM digestibility and nitrogen retention [4]. In fish studies, LPC supplementation did not significantly affect EE digestibility [18]. Further research is warranted to investigate the digestibility of EE in diets incorporating LPC in greater detail.

LPC not only enhances nutrient digestibility, but also improves intestinal morphology. Brautigan et al [19] reported that the addition of LPC to diets containing a combination of animal and vegetable fats increased the villus height (VH) in the jejunum. Boontiam et al [12] demonstrated that LPC supplementation increases VH and the ratio of VH to crypt depth in broiler chickens. Papadopoulos et al [20] observed that dietary LPC increased mucosal height. It has been proposed that the enhancement in growth performance and feed utilization can be attributed to the increased surface area between nutrients and intestinal villi [21]. Therefore, improved growth performance following LPC supplementation is likely linked to increased nutrient digestibility and improved gut epithelial morphology.

### Carcass traits

Carcass yield is a critical economic indicator and pivotal performance metric in the context of chicken slaughterhouses. In the current study, the incorporation of LPC resulted in a decrease in abdominal fat deposition and exhibited a tendency to increase carcass weight. These findings align with those of previous reports, which indicated that carcass weight and yield were significantly enhanced, while abdominal fat decreased in broilers treated with compound emulsifiers [22]. It can be hypothesized that the addition of an emulsifier could potentially lead to improved nutrient digestibility and redirection of its utilization towards growth and meat production, rather than inefficient metabolic processes such as fat deposition. The observed improvements in the digestibility of DM and CP in our study support this hypothesis.

Furthermore, a noteworthy reduction in liver weight was observed following the inclusion of LPC, which is consistent with the findings of Ghazalah et al [22]. Since LPC contributed to a reduction in liver fat content when broilers were fed diets with normal metabolic energy, it can be inferred that the decrease in liver weight was associated with a reduction in liver fat content. This suggests a reduced risk of fatty liver disease in broilers.

### Serum attribute

In our study, the serum concentrations of NEFA on day 21 and TG and NEFA on day 42 were all decreased by dietary LPC supplementation, which is consistent with the findings reported by Jansen et al [4]. Additionally, Zhao et al [16] demonstrated a reduction in the TG concentration due to LPC supplementation on day 14. The lower serum levels of TG and NEFA in broilers fed LPC may be attributed to the faster absorption and metabolism of dietary fat.

However, studies investigating the effects of dietary emulsifiers on blood parameters have yielded inconsistent results. In the current study, serum TC, TG, and GLU concentrations on day 21, as well as serum TC and GLU concentrations on day 42, were not significantly affected by the inclusion of LPC. Similarly, Park et al [23] reported that LPC inclusion did not significantly affect serum TC, TG, or NEFA concentrations. In juvenile turbot, dietary LPC was found to decrease the serum concentrations of TG and TC [18]. Therefore, the mechanism underlying the influence of emulsifiers on serum profiles remains a subject that requires further study and investigation.

### Liver health and antioxidant capacity

Serum ALT and AST activities serve as important markers of liver health, as they are primarily distributed in the plasma of hepatic cells and are released into the bloodstream when hepatocytes are damaged [24]. Weng et al [25] reported that dietary addition of LPC decreased the activities of ALT and AST, which aligns with the findings of the present study. In our study, the activity of ALT on day 21 was significantly reduced by dietary LPC, and there was a tendency towards lower AST activity on day 42. These results suggest that LPC has a positive effect on liver health.

Antioxidant capacity is a widely used indicator for evaluating physical conditions [26]. MDA is a product of lipid peroxidation and has detrimental effects on the structure and function of cellular membranes. Its concentration indirectly indicates the extent of lipid peroxidation damage [27]. In our study, LPC significantly reduced MDA content in the liver when broilers were fed diets with lower ME. These findings are supported by previous reports in juvenile large yellow croaker, where 0.2% dietary LPC was found to significantly reduce the MDA content [25]. It has been demonstrated that LPC enhances liver homeostasis by promoting its antioxidant capacity, contributing to improved liver health.

### Immune function

Cytokines play a pivotal role in the inflammatory response process [28]. Among these, IL-1β and IL-6, known as potent proinflammatory cytokines, are involved in most inflammatory conditions [29]. ET is a soluble complex of lipopolysaccharides found in the outer membrane of Gram-negative bacteria. It can induce a systemic inflammatory response by triggering the production of cytokines such as IL-6, IL-1, and tumor necrosis factor-α (TNF-α) [30].

In the current study, the inclusion of LPC led to a significant reduction in serum IL-6 levels (Tables 8 and 9) and resulted in a lower ET content in the serum of broilers (Table 9). To the best of our knowledge, this is the first report of decreased IL-6 and ET levels regulated by LPC in broilers. In juvenile large yellow croaker, LPC supplementation significantly downregulated the mRNA expression of TNF-α and IL-1β [25]. These findings collectively demonstrate that LPC can inhibit inflammatory responses and promote overall health.

## CONCLUSION

In conclusion, LPC supplementation of broiler diets can result in several beneficial effects. It decreases the FCR from days 1 to 42, increases the apparent digestibility of DM, and reduces the percentage of relative liver and abdominal fat. Moreover, LPC promotes liver health, improves antioxidant capacity, and enhances immunity. Importantly, LPC exerts these positive effects in broilers fed diets with both low and normal energy levels.

## Figures and Tables

**Table 1:** Ingredients and composition of the experimental diets

Items	Day 1 to 21		Day 22 to 42	
	
	PC^1)^	NC	PC	NC
Ingredients (%)				
Corn (7.8% CP)	50.39	50.85	55.20	55.84
Soybean meal (46% CP)	35.65	36.38	30.50	30.86
Corn gluten meal (60% CP)	4.00	3.80	3.30	3.30
Sodium chloride	0.28	0.28	0.28	0.28
Limestone (37%)	1.75	1.75	1.62	1.62
CaHPO_4_ type III	1.55	1.55	1.40	1.40
Soybean oil	4.60	3.60	6.00	5.00
Vitamin premix^1)^	0.05	0.05	0.05	0.05
Trace element premix^2)^	0.20	0.20	0.20	0.20
Choline chloride (50%)	0.10	0.10	0.10	0.10
DL-Methionine (98%)	0.35	0.35	0.35	0.35
L-Lysine HCL (98%)	0.80	0.80	0.75	0.75
Threonine (98.5%)	0.29	0.29	0.25	0.25
Total	100.00	100.00	100.00	100.00
Nutrient levels^3)^				
Metabolizable energy (kcal/kg)	3,070	2,980	3,190	3,100
Crude protein (%)	23.50	23.50	21.00	21.00
Calcium (%)	1.00	1.00	0.90	0.90
Available phosphorus (%)	0.50	0.50	0.48	0.48
Lysine (%)	1.39	1.39	1.30	1.30
Methionine+cysteine(%)	1.02	1.02	0.96	0.96
Threonine (%)	0.95	0.95	0.90	0.90

CP, crude protein.

1) PC, positive control; NC, negative control.

1) Vitamin premix provided per kg of complete diet: The premix provided the following per kg of diets: vitamin A (retinylacetate), 10,000 IU; vitamin D_3_ (cholecalciferol), 2,600 IU; vitamin E (DL-a-tocopherol acetate), 20 IU; vitamin K_3_ (menadione sodium bisulfate), 2 mg; vitamin B_1_ (thiamine mononitrate), 1.6 mg; vitamin B_2_ (riboflavin), 6 mg; vitamin B_6_ (pyridoxine hydrochloride), 3 mg; vitaminB_12_ (cyanocobalamin), 0.014 mg; nicotinic acid, 30 mg; pantothenic acid, 20 mg; biotin, 0.12 mg; folic acid, 0.8 mg; choline, 500 mg.

2) Mineral premix provided per kg of complete diet: iron, 80 mg; copper, 8 mg; manganese, 100 mg; zinc, 80 mg; iodine, 0.35 mg; selenium, 0.15 mg.

3) Calculated value.

**Table 2:** Effects of lysolecithins (LPC) and energy (ME) on growth performance of broiler chickens

ME	LPC (mg/kg)	BW (g)		ADG (g/d)			FCR		
		
		21 d	42 d	1–21 d	22–42 d	1–42 d	1–21 d	22–42 d	1–42 d
Normal	0	843.99	2,675.00	38.23	90.58	67.07	1.31	1.71	1.58
Normal	300	843.47	2,728.72	38.05	90.48	70.32	1.25	1.72	1.53
Low	0	802.75	2,677.01	36.20	89.13	69.67	1.34	1.73	1.56
Low	300	819.16	2,760.42	36.89	90.56	70.86	1.36	1.69	1.51
SEM	8.40	23.96	0.43	0.899	0.639	0.01	0.017	0.011	
Main effect									
ME level									
Normal	843.73	2,701.86	38.14	90.53	68.70	1.28	1.72	1.56	
low	810.96	2,718.71	36.54	89.85	70.27	1.35	1.71	1.54	
LPC (mg/kg)									
0	823.37	2,676.00	37.21	89.86	68.37	1.33	1.72	1.57	
300	831.31	2,744.57	37.47	90.52	70.59	1.31	1.70	1.52	
Significance									
ME level	0.07	0.73	0.08	0.710	0.244	0.011	0.706	0.443	
LPC	0.64	0.18	0.77	0.717	0.108	0.541	0.558	0.050	
ME×LPC	0.62	0.76	0.62	0.679	0.436	0.113	0.352	0.930	

BW, body weight; ADG, average daily gain; FCR, feed conversion ratio; SEM, standard error of the mean.

Note: In the same rank, values with different letter superscripts mean significant difference (p<0.05), while the same letter or no letter means no significant difference. A tendency was defined at “0.05<p<0.1”.

**Table 3:** Effects of lysolecithins (LPC) and energy (ME) on the apparent digestibility of nutrients in the terminal ileum of 42-day-old broilers

ME (kcal/kg)	LPC (mg/kg)	DM (%)	EE (%)	CP (%)	ADF (%)	NDF (%)
Normal	0	71.70^b^	62.02	55.14	35.10	39.89
	300	74.69^a^	61.75	58.12	35.07	42.95
Low	0	71.24^b^	61.05	55.93	29.22	36.36
	300	73.21^ab^	63.63	59.40	35.78	40.72
SEM		0.004	0.009	0.008	0.016	0.026
Main effect						
ME	Normal	73.20	61.89	56.63	35.09	41.42
	Low	72.23	62.34	57.67	32.50	38.54
LPC	0	71.47	61.54	55.54	32.16	38.13
	300	73.95	62.69	58.76	35.43	41.84
p-value	ME	0.288	0.811	0.537	0.422	0.584
	LPC	0.013	0.550	0.065	0.313	0.483
	ME×LPC	0.574	0.463	0.885	0.309	0.901

DM, dry matter; EE, ether extract; CP, crude protein; ADF, acid detergent fiber; NDF, acid detergent fiber; SEM, standard error of the mean.

a,b Means in same column with no common superscript differ significantly (p<0.05).

A tendency was defined at “0.05<p<0.1”.

**Table 4:** Effects of lysolecithins (LPC) and energy (ME) on organ relative weights of 42-day-old broilers

ME (kcal/kg)	LPC (mg/kg)	Carcass weight (%)	Abdominal fat (%)	Liver (%)
Normal	0	92.21	1.89	2.64^a^
	300	94.10	1.58	2.28^b^
Low	0	91.62	1.63	2.30^b^
	300	93.52	1.35	2.34^b^
SEM		0.005	0.001	0.000
Main effect				
ME	Normal	93.15	1.74	2.46
	Low	92.57	1.49	2.32
LPC	0	91.91	1.76	2.47
	300	93.81	1.47	2.31
Significance				
p-value	ME	0.577	0.083	0.065
	LPC	0.083	0.042	0.042
	Energy×LPC	0.995	0.923	0.015

SEM, standard error of the mean.

a,b Means in same column with no common superscript differ significantly (p<0.05).

**Table 5:** Effects of lysolecithins (LPC) and energy (ME) on serum parameters of 21-day-old broilers

ME (kcal/kg)	LPC (mg/kg)	GLU (mmol/L)	TG (mmol/L)	TC (mmol/L)	NEFA (μmol/mL)	AST (U/L)	ALT (U/L)
Normal	0	10.44	0.28	2.70	0.84	370.94	244.78^a^
	300	10.60	0.21	2.64	0.62	387.33	223.59^c^
Low	0	9.18	0.23	2.84	0.88	381.77	232.83^bc^
	300	9.67	0.17	2.80	0.64	373.02	235.90^ab^
SEM	0.381	0.027	0.073	0.026	3.984	1.794	
Main effect							
ME	Normal	10.52	0.25	2.67	0.73	379.14	234.18
	Low	9.43	0.20	2.82	0.76	377.40	234.62
LPC	0	9.81	0.26	2.77	0.86	376.36	238.80
	300	10.14	0.19	2.72	0.63	380.18	229.74
p-value	ME	0.166	0.424	0.317	0.555	0.830	0.961
	LPC	0.677	0.232	0.716	<0.001	0.639	0.022
	ME×LPC	0.836	0.928	0.952	0.926	0.136	0.004

Data were expressed as the mean±standard error (n = 6).

GLU, blood glucose; TG, triglyceride; TC, total cholesterol; NEFA, nonestesterified fatty acid; AST, aspartate aminotransferase; ALT, alanine aminotransferase; SEM, standard error of the mean.

a–c Different superscripts in the same column indicate significant differences (p<0.05).

**Table 6:** Effects of lysolecithins (LPC) and energy (ME) on serum parameters of 42-day-old broilers

ME (kcal/kg)	LPC (mg/kg)	GLU (mmol/L)	TG (mmol/L)	TC (mmol/L)	NEFA (μmol/mL)	AST (U/L)	ALT (U/L)
Normal	0	11.17	0.34	2.93	0.37	322.93	244.78
	300	11.53	0.27	2.80	0.18	313.53	294.78
Low	0	11.06	0.27	2.84	0.47	310.11	315.43
	300	10.95	0.23	2.51	0.25	267.88	286.27
SEM		0.152	0.013	0.085	0.027	6.436	2.183
Main effect							
ME	Normal	11.35	0.30	2.87	0.28	318.23	269.78
	Low	11.01	0.25	2.68	0.36	289.00	300.85
LPC	0	11.12	0.30	2.89	0.42	316.52	280.11
	300	11.24	0.25	2.66	0.22	290.71	290.53
p-value	ME	0.264	0.055	0.276	0.139	0.040	0.800
	LPC	0.695	0.041	0.196	0.001	0.066	0.200
	ME×LPC	0.447	0.640	0.575	0.832	0.225	0.374

Data were expressed as the mean±standard error (n = 6).

GLU, blood glucose; TG, triglyceride; TC, total cholesterol; NEFA, nonestesterified fatty acid; AST, aspartate aminotransferase; ALT, alanine aminotransferase; SEM, standard error of the mean.

**Table 7:** Effects of lysolecithins (LPC) and energy (ME) on the liver parameters of 42-day-old broilers

ME (kcal/kg)	LPC (mg/kg)	TG (mmoL/L)	TC (mmoL/L)	SOD (U/mL)	MDA (nmol/mL)	GSH-Px (U/mL)	CAT (U/mL)
Normal	0	0.14^a^	0.08	254.91	0.51^ab^	53.89	110.12
	300	0.12^b^	0.07	263.04	0.44^b^	53.89	95.45
Low	0	0.12^ab^	0.08	253.63	0.54^a^	49.47	118.28
	300	0.13^ab^	0.07	275.91	0.34^c^	58.09	112.30
SEM		0.004	0.006	9.610	0.013	1.320	4.447
Main effect							
ME	Normal	0.13	0.08	258.98	0.48	53.89	102.79
	Low	0.13	0.08	264.77	0.44	53.78	115.29
LPC	0	0.13	0.08	254.27	0.53	51.68	114.2
	300	0.12	0.07	269.48	0.39	55.99	103.88
p-value	ME	0.425	0.914	0.766	0.231	0.968	0.261
	LPC	0.188	0.299	0.438	<0.001	0.119	0.177
	ME×LPC	0.059	0.922	0.716	0.025	0.118	0.631

Data were expressed as the mean±standard error (n = 6).

TG, triglyceride; TC, total cholesterol; SOD, superoxide dismutase; MDA, malondialdehyde; GSH-Px, glutathione peroxidase; CAT, catalase; SEM, standard error of the mean.

a,b Different superscripts in the same column indicate significant differences (p<0.05).

**Table 8:** Effects of lysolecithins (LPC) and energy (ME) on serum immune indicators of 21-day-old broilers

ME (kcal/kg)	LPC(mg/kg)	ET (mmol/L)	IgA (mmol/L)	IgG (mmol/L)	IL-6 (mmol/L)	IL-1β (mmol/L)
Normal	0	62.90	244.06	1,956.11	27.18^ab^	455.67
	300	59.65	231.33	1,813.75	27.84^ab^	429.00
Low	0	59.77	232.81	1,979.72	30.15^a^	442.00
	300	62.84	244.20	1,940.83	26.67^b^	446.33
SEM		0.864	3.214	32.244	0.484	4.938
Main effect						
ME	Normal	61.28	237.70	1,884.93	27.51	442.34
	Low	61.31	238.51	1,960.28	28.41	444.12
LPC	0	61.34	238.44	1,967.92	28.67	448.84
	300	61.25	237.77	1,877.29	27.26	437.67
p-value	ME	0.984	0.902	0.261	0.366	0.857
	LPC	0.959	0.919	0.180	0.164	0.287
	ME×LPC	0.090	0.079	0.435	0.047	0.151

Data were expressed as the mean±standard error (n = 6).

ET, endotoxin; IgA, immunoglobulin A; IgG, immunoglobulin G; IL-1β, interleukin 1β; IL-6, interleukin 6; SEM, standard error of the mean.

a,b Different superscripts in the same column indicate significant differences (p<0.05).

**Table 9:** Effects of lysolecithins (LPC) and energy (ME) on serum immune indicators of 42-day-old broilers

ME (kcal/kg)	LPC (mg/kg)	ET (mmol/L)	IgA (mmol/L)	IgG (mmol/L)	IL-6 (mmol/L)	IL-1β (mmol/L)
Normal	0	68.68^a^	208.36	1,876.67	28.68	459.40
	300	56.43^b^	205.03	1,886.67	24.79	424.00
Low	0	58.56^b^	212.67	1,893.33	30.47	436.10
	300	55.98^b^	225.03	1,993.33	26.68	418.20
SEM		1.044	2.784	40.101	0.664	8.219
Main effect						
ME	Normal	62.55	206.69	1,881.67	26.74	441.70
	Low	57.27	218.85	1,943.33	28.58	427.15
LPC	0	63.62	210.51	1,885.00	29.58	447.75
	300	56.21	215.03	1,940.00	25.74	421.10
p-value	ME	0.021	0.041	0.454	0.186	0.388
	LPC	0.002	0.427	0.503	0.011	0.122
	ME×LPC	0.033	0.174	0.583	0.968	0.601

Data were expressed as the mean±standard error (n = 6).

ET, endotoxin; IgA, immunoglobulin A; IgG, immunoglobulin G; IL-1β, interleukin 1β; IL-6, interleukin 6; SEM, standard error of the mean.

a,b Different superscripts in the same column indicate significant differences (p<0.05).

## References

[1] Noy Y, Sklan D (1998). Metabolic responses to early nutrition. J Appl Poult Res.

[2] Gheisar MM, Hosseindoust A, Kim HB, Kim IH (2015). Effects of lysolecithin and sodium stearoyl-2-lactylate on growth performance and nutrient digestibility in broilers. Korean J Poult Sci.

[3] Wealleans AL, Jansen M, di Benedetto M (2020). The addition of lysolecithin to broiler diets improves growth performance across fat levels and sources: a meta-analysis of 33 trials. Br Poult Sci.

[4] Jansen M, Nuyens F, Buyse J, Leleu S, Van Campenhout L (2015). Interaction between fat type and lysolecithin supplementation in broiler feeds. Poul Sci.

[5] Zhang B, Li H, Zhao D, Guo Y, Barri A (2011). Effect of fat type and lysophosphatidylcholine addition to broiler diets on performance, apparent digestibility of fatty acids, and apparent metabolizable energy content. Anim Feed Sci Technol.

[6] Ahmad A, Mughal GA, Abro R (2023). Effect of lipase and lysolecithin supplementation with low energy diet on growth performance, biochemical attributes and fatty acid profile of breast muscle of broiler chickens. Animals (Basel).

[7] Wickramasuriya SS, Cho HM, Macelline SP (2020). Effect of calcium stearoyl-2 lactylate and lipase supplementation on growth performance, gut health, and nutrient digestibility of broiler chickens. Asian-Australas J Anim Sci.

[8] Arshad MA, Bhatti SA, Hassan I, Rahman MA, Rehman MS (2020). Effects of bile acids and lipase supplementation in low-energy diets on growth performance, fat digestibility and meat quality in broiler chickens. Braz J Poult Sci.

[9] McCarthy JF, Aherene FX, Okai DB (1974). Use of HCl insoluble ash as an index material for determining apparent digestibility with pigs. Can J Anim Sci.

[10] Niu ZY, Shi JS, Liu FZ, Wang XH, Gao CQ, Yao LK (2009). Effects of dietary energy and protein on growth performance and carcass quality of broilers during starter phase. Int J Poult Sci.

[11] Ge XK, Wang AA, Ying ZX (2019). Effects of diets with different energy and bile acids levels on growth performance and lipid metabolism in broilers. Poult Sci.

[12] Boontiam W, Jung B, Kim YY (2017). Effects of lysophospholipid supplementation to lower nutrient diets on growth performance, intestinal morphology, and blood metabolites in broiler chickens. Poult Sci.

[13] Khonyoung D, Yamauchi K, Suzuki K (2015). Influence of dietary fat sources and lysolecithin on growth performance, visceral organ size, and histological intestinal alteration in broiler chickens. Livest Sci.

[14] Boontiam W, Hyun YK, Jung B, Kim YY (2019). Effects of lysophospholipid supplementation to reduced energy, crude protein, and amino acid diets on growth performance, nutrient digestibility, and blood profiles in broiler chickens. Poult Sci.

[15] Øverland M, Tokach MD, Cornelius SG, Pettigrew JE, Rust JW (1993). Lecithin in swine diets: I. weanling pigs. J Anim Sci.

[16] Zhao PY, Kim IH (2017). Effect of diets with different energy and lysophospholipids levels on performance, nutrient metabolism, and body composition in broilers. Poult Sci.

[17] Honda K, Kamisoyama H, Isshiki Y, Hasegawa S (2009). Effects of dietary fat levels on nutrient digestibility at different sites of chicken intestines. J Poult Sci.

[18] Li BS, Li Z, Sun YZ, Wang SX, Huang BS, Wang JY (2019). Effects of dietary lysolecithin (LPC) on growth, apparent digestibility of nutrient and lipid metabolism in juvenile turbot Scophthalmus maximus L. Aquac Fish.

[19] Brautigan DL, Li R, Kubicka E (2017). Lysolecithin as feed additive enhances collagen expression and villus length in the jejunum of broiler chickens. Poult Sci.

[20] Papadopoulos GA, Poutahidis T, Chalvatzi S (2018). Effects of lysolecithin supplementation in low-energy diets on growth performance, nutrient digestibility, viscosity and intestinal morphology of broilers. Br Poult Sci.

[21] Taghavizadeh M, Shekarabi SPH, Mehrgan MS, Islami HR (2020). Efficacy of dietary lysophospholipids (Lipidol™) on growth performance, serum immuno-biochemical parameters, and the expression of immune and antioxidant-related genes in rainbow trout (Oncorhynchus mykiss). Aquaculture.

[22] Ghazalah A, Abd-Elsamee M, Ibrahim M (2021). Effects of a combination of lysolecithin, synthetic emulsifier, and monoglycerides on growth performance, intestinal morphology, and selected carcass traits in broilers fed low-energy diets. Animals.

[23] Park JH, Nguyen DH, Kim IH (2018). Effects of exogenous lysolecithin emulsifier supplementation on the growth performance, nutrient digestibility, and blood lipid profiles of broiler chickens. J Poult Sci.

[24] Lu CH (2015). Application research of substitution of emulsifier for partial oil in the production of broilers [master’s thesis]. Nanjing, China: Nanjing Agricultural Univ;.

[25] Weng M, Zhang W, Zhang Z (2022). Effects of dietary lysolecithin on growth performance, serum biochemical indexes, antioxidant capacity, lipid metabolism and inflammation-related genes expression of juvenile large yellow croaker (Larimichthys crocea). Fish Shellfish Immunol.

[26] Deng S, Fu A, Junaid M (2019). Nitrogen-doped graphene quantum dots (N-GQDs) perturb redox-sensitive system via the selective inhibition of antioxidant enzyme activities in zebrafish. Biomaterials.

[27] Ayala A, Muñoz MF, Argüelles S (2014). Lipid peroxidation: production, metabolism, and signaling mechanisms of malondialdehyde and 4-hydroxy-2-nonenal. Oxid Med Cell Longev.

[28] Mucksová J, Chalupský K, Plachý J (2018). Simultaneous detection of chicken cytokines in plasma samples using the Bio-Plex assay. Poult Sci.

[29] Ghareeb K, Awad WA, Soodoi C, Sasgary S, Strasser A, Böhm J (2013). Effects of feed contaminant deoxynivalenol on plasma cytokines and mRNA expression of immune genes in the intestine of broiler chickens. PLoS One.

[30] Benson S, Engler H, Wegner A (2017). What makes you feel sick after inflammation? predictors of acute and persisting physical sickness symptoms induced by experimental endotoxemia. Clin Pharmacol Ther.

